# Systematic review and meta-analysis of endovascular therapy versus open surgical repair for the traumatic lower extremity arterial injury

**DOI:** 10.1186/s13017-024-00544-9

**Published:** 2024-04-27

**Authors:** Yuhan Qi, Jiarong Wang, Ding Yuan, Pengchao Duan, Li Hou, Tiehao Wang

**Affiliations:** 1 Division of Vascular Surgery Department of General Surgery, West China Hospital, Sichuan University, 37 Guo Xue Alley, Chengdu, Sichuan Province 610041 China; 2 West China School of Medicine, West China Hospital, Sichuan University, Chengdu, China

**Keywords:** Endovascular therapy, Open surgical repair, Traumatic lower extremity arterial injury, Amputation, Meta-analysis

## Abstract

**Objective:**

For traumatic lower extremity artery injury, it is unclear whether it is better to perform endovascular therapy (ET) or open surgical repair (OSR). This study aimed to compare the clinical outcomes of ET versus OSR for traumatic lower extremity artery injury.

**Methods:**

The Medline, Embase, and Cochrane Databases were searched for studies. Cohort studies and case series reporting outcomes of ET or OSR were eligible for inclusion. Robins-I tool and an 18-item tool were used to assess the risk of bias. The primary outcome was amputation. The secondary outcomes included fasciotomy or compartment syndrome, mortality, length of stay and lower extremity nerve injury. We used the random effects model to calculate pooled estimates.

**Results:**

A total of 32 studies with low or moderate risk of bias were included in the meta-analysis. The results showed that patients who underwent ET had a significantly decreased risk of major amputation (OR = 0.42, 95% CI 0.21–0.85; I^2^=34%) and fasciotomy or compartment syndrome (OR = 0.31, 95% CI 0.20–0.50, I^2^ = 14%) than patients who underwent OSR. No significant difference was observed between the two groups regarding all-cause mortality (OR = 1.11, 95% CI 0.75–1.64, I^2^ = 31%). Patients with ET repair had a shorter length of stay than patients with OSR repair (MD=-5.06, 95% CI -6.76 to -3.36, I^2^ = 65%). Intraoperative nerve injury was just reported in OSR patients with a pooled incidence of 15% (95% CI 6%–27%).

**Conclusion:**

Endovascular therapy may represent a better choice for patients with traumatic lower extremity arterial injury, because it can provide lower risks of amputation, fasciotomy or compartment syndrome, and nerve injury, as well as shorter length of stay.

**Supplementary Information:**

The online version contains supplementary material available at 10.1186/s13017-024-00544-9.

## Introduction

Although the incidence of traumatic lower extremities arterial injuries ranged from 0.3 to 0.4% in trauma patients in the United States [1, 2], these arterial injuries could lead to an amputation rate as high as 16.2% [3, 4]. Timely and effective vascular repair plays a vital role in saving limbs and lives, but first-line revascularization strategy remains controversial.

As current guidelines have not covered evidence-based decision making for optimal revascularization procedure for traumatic artery injury [5, 6], an increasing number of publications emerged reporting outcomes based on their center experience [2, 7–9] Open surgical repair (OSR) remained as the classic standard procedure for traumatic injuries to the lower extremity arteries with high limb salvage rates [9–11]. However, OSR is sometimes accompanied with larger surgical wounds, longer operating time and potentially higher wound complications [12]. Meanwhile, with fast advances in endovascular equipment, endovascular therapy (ET) has been serving as one of the major procedures for vascular injuries in the past two decades [7, 13, 14], especially in blunt trauma patients [13]. Compared to OSR, ET may have potential advantages in the aspect of prompt control of bleeding without vessel exposure and nerve injury, minimally invasive wound, and shorter operating time [11, 13]. However, whether OSR or ET provides better postoperative outcomes for patients with traumatic lower extremity arterial injury remains inconclusive and ought to be further elucidated.

By summarizing all available evidence, the present systematic review and meta-analysis aimed to compare postoperative outcomes of patients who underwent OSR versus ET for traumatic lower extremity arterial injury, hoping to aid in establishment of evidence-based decision making.

## Materials and methods

### Review design

The present systematic review and meta-analysis was conducted according to the Meta-analyses Of Observational Studies in Epidemiology (MOOSE) standards [15]. The present study has been reported in line with PRISMA (Preferred Reporting Items for Systematic Reviews and Meta-Analyses) and AMSTAR (Assessing the methodological quality of systematic reviews) Guidelines [16, 17]. This protocol was registered in PROSPERO (CRD42021289629).

### Literature search

Systematic searches were conducted in the following databases from inception through to January 14, 2023, without language restrictions: MEDLINE, EMBASE, and Cochrane Central Register of Controlled Trials for randomized controlled trials. The keywords of searching terms are listed as follows: type of intervention: ‘‘bypass’’, ‘‘open’’, ‘‘endovascular’’, ‘‘angioplasty’’, and ‘‘stenting’’; interested disease: ‘‘lower extremity’’ and “artery injury”. The details of the search strategy are available in Appendix 1.

### Study selection and criteria for consideration of studies

Titles, abstracts, and full-text publications were independently screened by two authors (YQ and JW), and the prespecified inclusion criteria were as follows: (1) clinical studies reporting outcomes of patients with traumatic lower extremity artery injuries below the inguinal ligament, including injuries to the iliac artery; (2) studies reported outcomes after ET or/and OSR of traumatic artery injuries, outcomes included survival, amputation, compartment syndrome, and other related outcomes; (3) The study design included randomized controlled trials, cohort studies, or case series studies with a sample size of more than 10 participants. Studies were excluded if no specific data can be extracted for ET or OSR treatment, or if a duplicate cohort was included. Disagreements were resolved through discussion with a third reviewer (DY).

### Data collection, extraction, and outcomes of interest

Two independent researchers (YQ and JW), blind to each other, collected and extracted data using a predefined extraction form the eligible studies, and any discrepancies were checked and resolved with a third reviewer (TW). The following baseline characteristics of included patients were collected: age, sex, number of patients, study design, lesion site, injury type (blunt, penetrating, or unspecified), and intervention details (endovascular therapy was defined as stent placement or percutaneous transluminal angioplasty (PTA), while open surgical repair included suture, incision, bypass, ligition and patch techniques). The primary outcome of interest was the rate of postoperative major amputation (major amputation defined as above ankle amputation). Secondary outcomes included fasciotomy or compartment syndrome (defined as a combination event of fasciotomy or compartment syndrome), all-cause mortality, length of stay (LOS), and nerve injury.

### Risk of bias assessment

Based on the new version of the Cochrane handbook for systematic review and meta-analysis, the risk of bias was independently assessed by two reviewers (YQ and JW) with the Robins-I tool for non-randomized studies [18]. An 18-item tool was used to assess the quality of the case series. Disagreements were discussed and resolved by a third reviewer (DY or TW).

## Statistical methods

We performed the data analyses using Review Manager Version 5.3 (The Nordic Cochrane Centre, København, Denmark) and STATA 14.1 (StataCorp, College Station, TX, USA) based on methods described in the Cochrane Handbook for Systematic Reviews of Interventions (version 6.2). For continuous variables, means and standard deviations were used to pool the overall estimates, and if means and standard deviations were unavailable, the method introduced by Hozoet al. [19] was applied for conversion. For dichotomous data, we calculated odds ratio (OR) and 95% confidence intervals (CIs) with the Mantel–Haenszel method [20]. Statistical heterogeneity across studies was estimated using the I^2^ statistic, and an I^2^ value larger than 75% indicates high heterogeneity [21]. To further explore the potential source of heterogeneity, post hoc meta-regression analyses were performed for the outcomes of high heterogeneity. Funnel plots were conducted to assess publication bias of the included studies. We also performed subgroup analyses stratified by injured artery and injury type. all outcomes were showed in Table 1.

**Table 1:** Summary of findings for ET and OSR in traumatic lower extremity arterial injury

	ET	OSR	ET vs. OSR
Outcomes	Proportion (%)	Proportion (%)	Effect measure
Major amputation	3%	9%	0.42* (0.21–0.85)
Iliofemoral	4%	9%	0.15* (0.05–0.45)
Popliteal	5%	11%	-
Penetrating injury	NA	5%	-
Injury with fracture	3%	10%	-
Fasciotomy or compartment syndrome	9%	23%	0.31* (0.20–0.50)
Compartment syndrome	3%	8%	0.36* (0.25–0.50)
Nerve injury	0%	15%	-
Mortality	4%	2%	1.11 (0.75–1.64)
LOS	NA	NA	-0.56* (-6.67 to -3.36)
Penetrating injury	NA	NA	-6.12* (-7.21 to -5.03)

ET = endovascular therapy; OSR = open surgical repair; LOS = length of stay; *p < 0.05

## Results

### Description of included studies

**Fig. 1:**
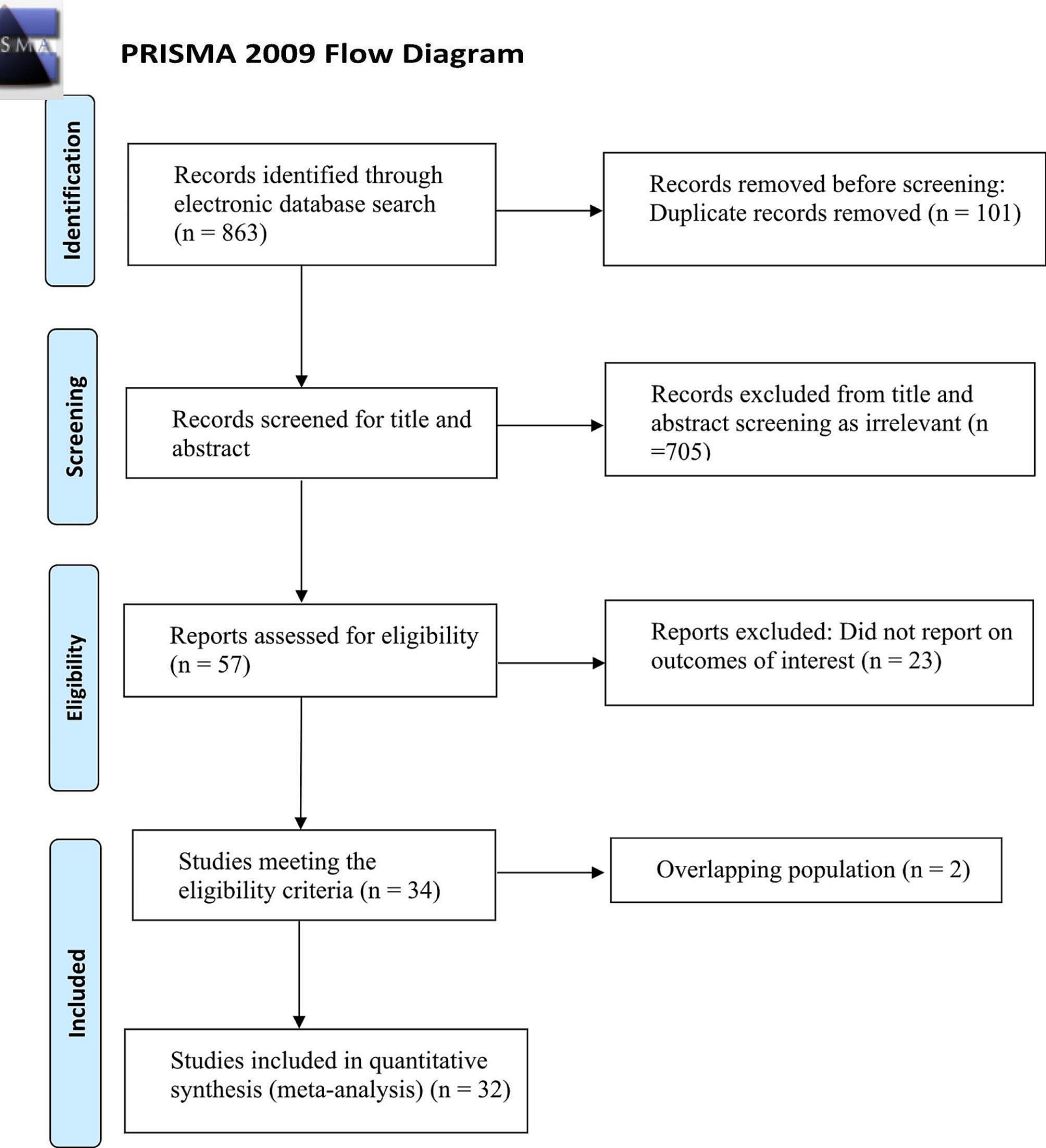
Study selection flow diagram

After literature search, a total of 863 records were identified through the electronic database search. After duplicates were removed, 762 potential publications were left for further assessment. The literature selection generated 34 articles, of which two publications [22, 23] had partially overlapping cohorts derived from the same database (The National Trauma Data Bank). We only included Potter’s study because it had larger sample size with propensity score matching. Finally, 32 studies (10 cohorts [13, 22–30]and 22 case series [8, 10, 12, 31–49] of 1577 ET and 6097 OSR patients were included in the quantitative analysis. The PRISMA flow diagram is presented in Fig. 1. Eleven studies [8, 10, 28, 29, 32–34, 40, 42, 43, 45] reported outcomes of popliteal artery injury. As for injury types, six articles [12, 25, 30, 31, 39, 40] reported penetrating injuries and four articles [25, 37, 40, 43] reported blunt injuries, others involved mixed types of injury. Study characteristics are presented in Table 2 and Supplemental Table 1.

**Table 2:** Characteristics of included studies

Study, Year	Design	Population	Simple size-n (ET/OSR)	Target site	Traumatic type §	Mean age-y (ET/OSR)	Male-n (ET/OSR)
Potter, 2021 ^23^	R	Adult	157/628	iSFA/PA/SFA/PA	B + P	40/39	125/503
Abdou(P) †, 2021 ^25^	R	Adult	198/198	IA	P	28/27.33	179/184
Abdou(B)‡, 2021 ^25^	R	Adult	335/335	IA	B	45/43.67	196/196
Degmetich, 2020 ^22^	P	Both	390/1865	SFA	B + P + U	44.9/32.5	285/1660
Ratnasekera, 2020 ^30^	R	Adult	30/18	iSFA/PA	P	25.33/28.67	26/18
Maithel, 2020 ^29^	R	Pediatric	6/31	PA	B + P	16/15	6/26
Butler, 2019 ^27^	R	Adult	37/456	SFA	B + P + U	50.9/36.3	22/387
Wahab, 2019 ^24^	R	Adult	10/10	FA	NA	28.5/27.5	10/10
Branco, 2017 ^26^	R	Pediatric	120/616	CFA/SFA/PA/ATA/PTA	NA	NA	NA
Branco, 2014 ^13^	R	NA	128/128	CFA/SFA/PA/ATA/PTA	NA	NA	NA
Dua, 2014 ^28^	R	NA	19/24	PA	NA	NA	NA
Banion, 2021 ^42^	C	Adult	NA/302	PA	B + P	NA/32	239/302
Jiang, 2021 ^8^	C	Adult	46/NA	iPA	B + P	50.6/NA	32/NA
Hundersmarck, 2021 ^37^	C	NA	NA/16	PA	B	NA	NA
Georgakarakos, 2021 ^36^	C	Adult	17/NA	CIA/EIA/CFA/SFA/PA/ATA/PTA	B + P	NA	NA
Asensio, 2020 ^32^	C	Both	NA/76	PA	B + P	NA/29	NA/58
Rehman, 2020 ^45^	C	Adult	NA/40	PA	B + P	NA/32	NA/100
Sharrock, 2019 ^12^	C	NA	NA/596	CIA/EIA/CFA/SFA/PFA/PA/ ATA/PTA/ PA†	P	NA/25.19	NA
Prieto, 2019 ^44^	C	Pediatric	NA/42	PTA /ATA	NA	NA	NA
Magnotti (P)†, 2020^40^	C	Adult	NA/123	PA	P	NA/32	NA/116
Magnotti (B)‡, 2020^40^	C	Adult	NA/91	PA	B	NA/38	NA/69
Mousa, 2018 ^41^	C	Pediatric	NA/76	SFA/PA/PTA	B + P	NA	NA
Şahin, 2018 ^46^	C	Adult	NA/21	NA	NA	NA	NA
Kufner, 2015 ^39^	C	Adult	30/NA	IA/ PA	NA	71/NA	36.7/NA
Lang, 2015 ^10^	C	NA	NA/64	PA	NA	NA/44	NA/44
Dua, 2014 ^34^	C	Adult	NA/64	PA	NA	NA/28	NA/28
Sciarretta, 2014 ^47^	C	Pediatric	NA/32	PA/ATA/PTA/ PA*	B + P	NA /14.7	NA /17
Bernhoff, 2013 ^33^	C	Adult	NA/31	PA	NA	NA	NA
Dua, 2012 ^35^	C	NA	NA/72	CFA/SFA/PA/TA	NA	NA	NA
Trellopoulo-s, 2012 ^48^	C	NA	NA/13	NA	NA	NA	NA
Pourzand, 2010 ^43^	C	Both	NA/72	PA	B	NA/34	NA/70
Huynh, 2006 ^38^	C	Both	NA/57	FA /PA	B + U	NA/31	NA/44
White, 2006 ^49^	C	NA	44/NA	IA/ FA	NA	NA	NA
Aksoy, 2005 ^31^	C	NA	10/NA	NA	P	21.58/NA	NA

Data are expressed as mean +- standard deviation or median (range) or mean; a Data are expressed as median; NA = not available

IA= iliac artery; CIA = common iliac artery; EIA = external iliac artery; CFA = common femoral artery; SFA = superficial femoral artery; iSFA = isolated superficial femoral artery; TA = tibial artery; PFA = profunda femoral artery; PA = popliteal artery; ATA = anterior tibial artery; PTA = posterior tibial artery; TA = tibial artery; PA*= Peroneal artery; † penetrating injury; ‡ blunt injury; R = Retrospective; P = Prospective; C = case series; § B = blunt; P = penetrating; U = unspecified

### Risk of bias of included studies

The results of the ROBINS-1 tool for cohort studies showed low to moderate risk of bias in most domains of the included studies. Most of the included case series were assessed to be of moderate or high quality, and few were of low quality. The results of quality assessment are shown in Supplemental Tables 2 and Supplemental Table 3.

### Amputation

**Fig. 2:**
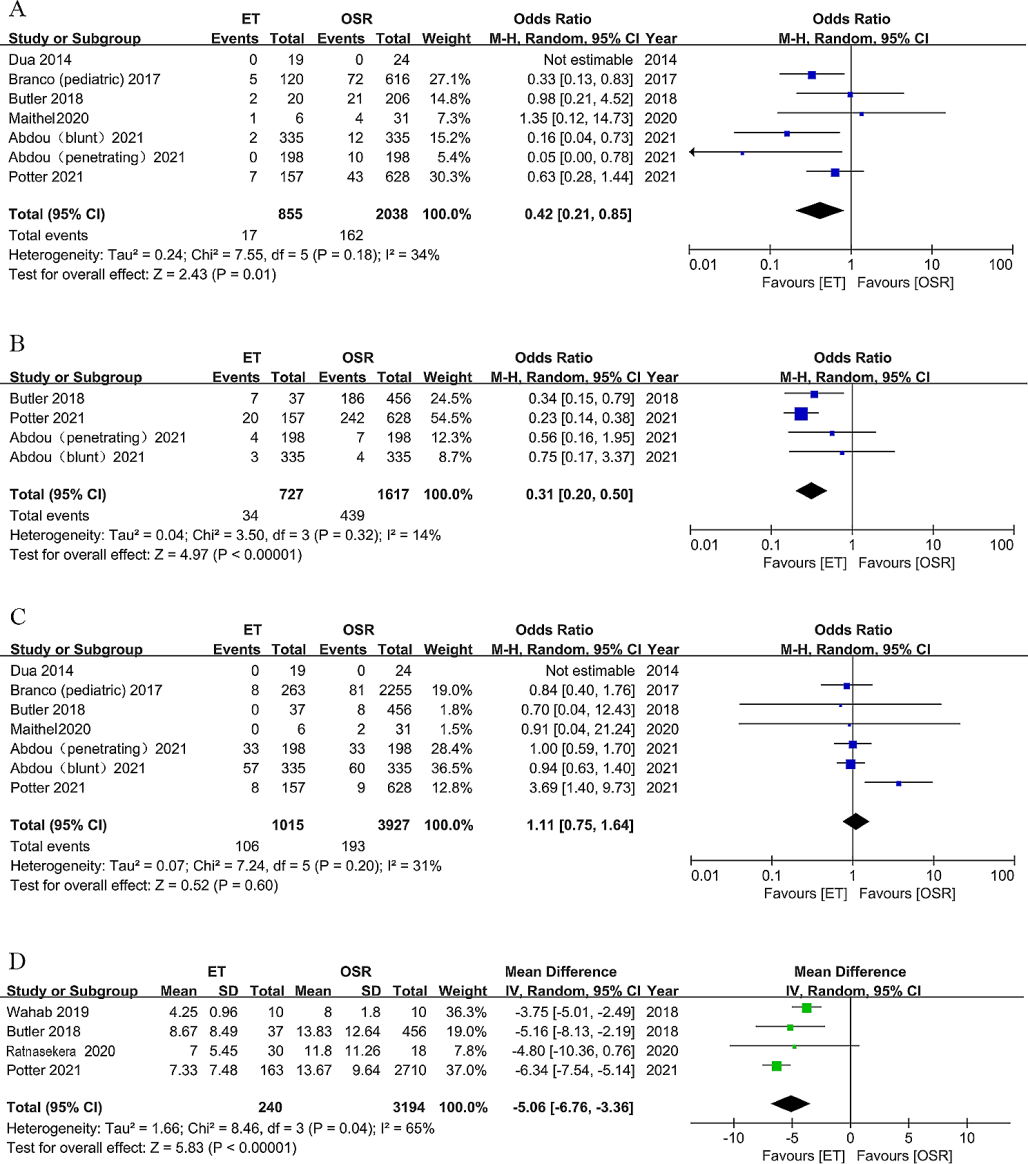
Forest plot of (**A**) studies for the difference in major amputation, (**B**) studies for difference in fasciotomy or compartment syndrome, (**C**) studies for the difference in mortality, and (**D**) studies for the difference in length of stay in patients with traumatic lower extremity arterial injury comparing ET vs. OSR. M-H = Mantele-Haenszel; CI = confidence interval; IV = inverse variance; ET = endovascular therapy; OSR = open surgical repair

**Fig. 3:**
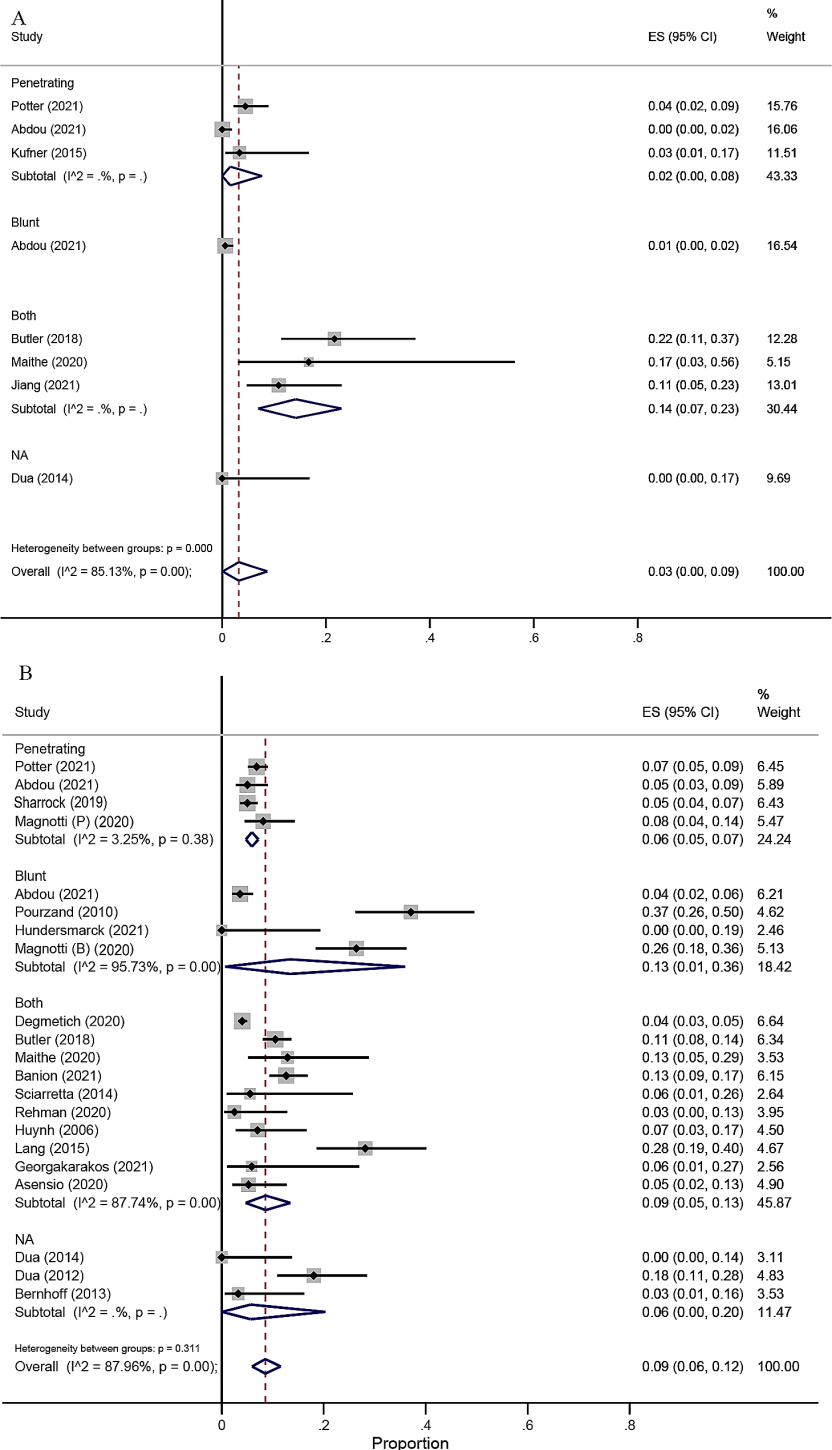
The pooled estimate for amputation in ET (**A**) and OSR (**B**) in patients with traumatic lower extremity arterial injury. ES = estimate proportions; CI = confidence interval; ET = endovascular therapy; OSR = open surgical repair

A total of 19 articles, comprising 2893 patients, reported the major amputation rate after endovascular or open surgical repair of traumatic artery injuries of lower limbs. Data from studies conducted by Branco et al. [26], Abdou et al. [25], and Potter [23] et al. were adjusted for propensity score matching. The pooled results from seven cohort studies suggested that patients who underwent ET had a significantly decreased risk of major amputation than patients who underwent OSR. (OR = 0.42, 95% CI 0.21–0.85; I^2^ = 34%, Fig. 2A). The pooled incidence major amputation rate was 3% (95% CI 0%–9%; I^2^ = 85.13%, Fig. 3A) in 828 ET patients and 9% (95% CI 6%–12%; I^2^ = 87.96%, Fig. 3B) in 5102 OSR patients. The pooled incidence major amputation rate was 3% (95% CI 0%–9%; I^2^ = 86.40%, Supplemental Fig. 1A) in ET adults and 8% (95% CI 5%–12%; I^2^ = 81.10%, Supplemental Fig. 1B) in OSR adults. ET had a significantly decreased risk of major amputation than patients who underwent OSR in adults. (OR = 0.47, 95% CI 0.27–0.80; I^2^ = 34%, Supplemental Fig. 1C).

Among the included studies, a total of 3 studies from 4 cohorts underwent propensity score matching analysis. Subgroup analysis of the propensity score-matched data revealed that patients undergoing ET had significantly lower major amputation risks compared to those undergoing OSR (OR = 0.32, 95% CI 0.14–0.72; I^2^ = 42%, Supplemental Fig. 1D). We conducted a meta-analysis using all available data from the included studies, comprising a total of 6623 patients. The results indicate that patients undergoing ET had a significantly lower risk of major amputation compared to those OSR (OR = 0.51, 95% CI 0.36–0.73; I^2^ = 0%, Supplemental Fig. 1E).

Subgroup analysis stratified by injured arteries suggested ET was also associated with a significantly decreased risk of amputation in patients with iliac or femoral arterial injury (OR = 0.15, 95% CI 0.05–0.45; I^2^ = 0%, Supplemental Fig. 1F), with an estimated amputation rate of 3% (95% CI 0%–12%; I^2^ = 92.49%) in ET group and 6% (95% CI 3%–9%; I^2^ = 88.43%) in OSR group. As for patients with isolated popliteal artery injury, the pooled results showed an estimated amputation rate of 5% (95% CI 0%–19%; I^2^ = 49.72%) in the ET group and 11% (95% CI 5%–18%; I^2^ = 85.32%) in OSR group.

In the subgroup of patients who suffered from penetrating artery injury, the results showed an estimated amputation rate of 5% (95% CI 4%–7%; I^2^ = 0.00%) in OSR group. In the subgroup of patients suffered from fracture, the results showed an estimated amputation rate of 10% (95% CI 7%–14%; I^2^ = 85.57%) in OSR group and 3% (95% CI 0%–10%; I^2^ = 91.25%) in ET group. Meta-regression analysis suggested that the pooled estimate for amputation was significantly associated with injury severity score (ISS) rather than fracture (ISS: t=-2.52, 95% CI 0.86–0.99, Supplemental Fig. 1G; fracture: t = 1.88, 95% Cl -0.88–7.94). in addition, the funnel plot on amputation rates appeared to be symmetrical on visual inspection, suggesting no publication bias, that could be confirmed statistically with the harbord’s linear regression test (*p* = 0.949) and egger’s linear regression test (*p* = 0.840).

### Fasciotomy or compartment syndrome, nerve injury

A total of 15 studies including 1163 ET patients and 4175 OSR patients reported outcomes of Fasciotomy or compartment syndrome. The pooled estimate of fasciotomy or compartment syndrome rate in the OSR subgroup (23%, 95% CI 13%–36%; I^2^ = 98.24%) was over two times higher than that of ET group (9%, 95% CI 3%–16%; I^2^ = 92.92%). In the further meta-analysis of four cohort studies, the results also revealed a significantly higher risk of fasciotomy or compartment syndrome in OSR than ET (OR = 0.31, 95% CI 0.20–0.50, I^2^ = 14%, Fig. 2B). We conducted a meta-analysis using all available data from the included studies, the results showed a significantly lower risk of fasciotomy or compartment syndrome in ET than OSR (OR = 0.44, 95% CI 0.26–0.74, I^2^ = 0%, Supplementary Fig. 2A). The pooled estimate of compartment syndrome rate in the OSR subgroup (8%, 95% CI 2%–17%; I^2^ = 0%) was over two times higher than that of ET group (3%, 95% CI 0%–10% I^2^ = 93.83%). In the further meta-analysis of cohort studies, the results also revealed a significantly higher risk of compartment syndrome in OSR than ET (OR = 0.36, 95% CI 0.25–0.50, I^2^ = 0%, Supplemental Fig. 2B). As for nerve injury, this complication was only reported in OSR patients, with an estimated rate of 15% (95% CI 6%–27%; I^2^ = 93.76%).

### Mortality and length of stay

Based on 1228 ET and 4285 OSR patients, the pooled estimate of postoperative mortality rate was 4% (95% CI 1%–9%; I^2^ = 87.58, Supplemental Fig. 3A) in the ET group and 2% (95% CI 1–4%; I^2^ = 58.67%, Supplemental Fig. 3B) in the OSR group. The in-hospital mortality results from the study by Butler et al. were included and meta-analysis of six cohorts revealed no significant difference in all-cause mortality between ET and OSR groups (OR = 1.11, 95% CI 0.75–1.64, I^2^ = 31%, Fig. 2C). We conducted a meta-analysis using all available data from the included studies, and the results showed no significant difference in all-cause mortality between ET and OSR groups (OR = 0.96, 95% CI 0.57–1.60, I^2^ = 82%, Supplementary Fig. 3C). Four cohort studies, comprising 3434 patients, reported the length of stay after ET or OSR. The pooled results suggested ET Patients had a significantly shorter length of stay than patients with OSR (MD=-5.06, 95% CI -6.76 to -3.36, I^2^ = 65%, Fig. 2D). Significant heterogeneity was noted, and we further performed subgroup analysis stratified by injury types. In patients with penetrating injury, similar results were observed with no heterogeneity (MD=-6.12, 95% CI -7.21 to -5.03, I^2^ = 0%, Supplemental Fig. 3D).

## Discussion

To the best of our knowledge, the present meta-analysis is the first systematic review and meta-analysis focusing on revascularization decision making in traumatic lower extremity arterial injury. Our findings suggested ET was associated with a significantly lower risk of amputation, fasciotomy or compartment syndrome, and nerve injury, as well as shorter length of stay, but no significant difference was found regarding postoperative all-cause mortality.

Since the advancement of endovascular interventions in the past several decades, more first-line treatment options emerged for traumatic arterial injury. Previous systematic and meta-analysis showed that endovascular therapy had a better clinical result for the traumatic ruptured thoracic aorta, including a lower risk of mortality and a satisfactory outcome in the duration of intensive care and total hospital stay, but with a higher rate of reintervention compared with open repair [50]. However, a summary of evidence regarding the treatment strategy for traumatic lower extremity arterial injury is lacking.

The incidence of limb amputation varies widely in lower extremity artery injury, ranging from 0 to 17% in ET patients and 3 to 18% in OSR patients [13, 22, 25, 27]. The present results showed that ET was associated with a significantly decreased risk of amputation than OSR, which might be attributed to shorter ischemic time of distal limbs from arrival to restoration of perfusion [43]. Previous studies showed that shortening the ischemic time as close to six hours as possible may lead to higher possibility of limb salvage [32, 51]. It was noteworthy that the severity of peripheral tissue damage was also a predictor of the risk of amputation [10], which would also determine the type of injured arteries (blunt or penetrating). Moreover, post-traumatic tissue infection in lower limb injuries is also a contributing factor to limb amputation. However, which severity score can predict lower extremity amputation in traumatic artery injury remained inconclusive [32]. In our present analysis, the results of meta-regression demonstrated a significant association between injury severity score (ISS) and the risk of low extremity amputation in patients. Unfortunately, we could not perform a subgroup analysis according to traumatic severity, as only a few studies reported detailed results of the injury severity score, and the present analysis cannot distinguish the risk of amputations which result from limb ischemia rather than tissue loss or infection. These distinctions would be meaningful if future studies could adjust the severity of trauma when comparing the postoperative outcomes of ET and OSR.

In addition to injury severity, injured site and involved artery were also important fact that affect the decision making of clinicians. Isolated popliteal artery injury has now become the focus among all injured arteries, for either endovascular or open repair is somewhat tactical and skillful, especially for the distal segment of the popliteal artery. Among included publications, the main open procedure for isolated popliteal artery injury was either end-to-end anastomosis or bypass, and the common endovascular procedure was the implantation of stent graft and percutaneous transluminal angioplasty. But concerns aroused regarding the long-term patency of stent graft near the knee joint. Previous studies indicated that the 6-month primary patency rate of traumatic popliteal artery injury is around 90% after OSR, and 82.9% after ET at 6 months. However, ET can have a 100% secondary patency rate at one year [8]. The results of our meta-analysis showed that the incidence of amputation after OSR (11%) was almost twice of that after ET (5%). Moreover, the incidence of amputation after OSR is more than three times as high as that after ET in patients with lower extremity fracture, though fracture might not predict amputation, which is similar to the previous results [51]. Despite the lower risk of postoperative amputation after ET, outcomes with longer follow-up are needed to verify the advantages of ET.

Compartment syndrome after tissue reperfusion could be a disturbing problem that can lead to irreversible necrosis of distal limbs. Some studies showed that ET could reduce the incidence of fasciotomy compared to OSR [23, 27], while some other studies did not observe such difference [22, 25]. Consistent with the results of amputation, our results suggested the risk of fasciotomy or compartment syndrome was higher in the OSR group compared to ET group. However, a possible bias may present, because fasciotomies may be performed prophylactically in the OSR patients, whereas the fasciotomies were more likely carried out on demands for therapeutic purposes on compartment syndrome in ET patients [8, 23]. Nevertheless, our results also showed that ET was associated with a lower risk of compartment syndrome, which further confirmed the findings regarding fasciotomy.

Prior studies demonstrated that Glasgow Coma Scale (GCS) score, Injury Severity Score (ISS), and systolic blood pressure (SBP) are independent predictors of death after arterial injury [26]. Recent study suggested that endovascular therapy could reduce 30-day and 1-year mortality in traumatic ruptured thoracic aorta compared to open surgical repair [50]. As for traumatic lower extremity arterial injury, potter et al. also demonstrated that OSR was associated with a higher risk of mortality [23], probably due to severer degree of trauma. Butler et al. shows that ET is associated with a higher risk of discharge mortality, which might be associated with a higher age and NISS scores among ET patients compared to OSR patients. Most included studies did not observe significant difference in mortality between two groups, which was consistent with the result of our meta-analysis. Likewise, the severity of injury should be taken into account when analyzing the results, which as limited in the current study due to lack of data.

### Limitation

Although this work is the largest meta-analysis regarding postoperative outcomes after ET versus OSR for lower extremity arterial injury, some limitations must be considered when interpreting our results. Firstly, the analysis was limited by the observational design of the included studies, most of which were retrospective, with the inherent limitation of selection bias. The second limitation relied on the concerns of significant heterogeneity in some results. To address this issue as much as possible, subgroup analyses were conducted and meta-regression was performed, meanwhile, in studies by Branco et al., Abdou et al., and Potter et al., we utilized propensity-matched data for analysis. Third, the severity of trauma was an important confounder of outcomes, but current data restricted us from performing further analysis. Even so, we applied meta-regression to demonstrate the association between ISS and the risk of amputation, which indicated that ISS may be utilized as a useful tool to adjust for the risk of amputation in future studies. Finally, Inconsistencies in treatment approaches across different studies may introduce certain biases. Future prospective cohort studies will help in evaluating the impact of specific treatment modalities on lower extremity arterial injuries.

## Conclusion

In conclusion, endovascular therapy could offer the advantages of lower risks of postoperative amputation, fasciotomy, or compartment syndrome, and nerve injury, as well as shorter length of stay, compared to OSR. But these two different procedures might not affect the risk of postoperative all-cause mortality. Although large prospective studies are needed to further confirm the long-term outcomes of ET, the results of this review may aid in the decision-making process of revascularization in patients with traumatic lower extremity artery injury.

### Electronic supplementary material

Below is the link to the electronic supplementary material.

Supplementary Material 1

Supplementary Material 2

Supplementary Material 3

## Data Availability

No datasets were generated or analysed during the current study.

## Abbreviations

ET: Endovascular therapy
OSR: Open surgical repair
MOOSE: Meta-analyses Of Observational Studies in Epidemiology
PRISMA: Preferred Reporting Items for Systematic Reviews and Meta-Analyses
AMSTAR: Assessing the methodological quality of systematic reviews
LOS: Length of stay
ISS: Injury Severity Score
GCS: Glasgow Coma Scale
