# Supplementary material for: Systematic review and meta-analysis of endovascular therapy versus open surgical repair for the traumatic lower extremity arterial injury
Source: World J Emerg Surg. 2024 Apr 27;19:16. doi: 10.1186/s13017-024-00544-9 (PMC11055329; doi:10.1186/s13017-024-00544-9)
Supplement: Supplementary file 3 — Supplementary Material 3 [file 13017_2024_544_MOESM3_ESM.docx]

**SUPPLEMENTARY MATERIALS**

**Appendix 1. Detailed search strategies**

**ovid medline(r) and epub ahead of print, in-process, in-data-review & other non-indexed citations, daily and versions(r) <1946 to january 14, 2023>**

1 exp Vascular System Injuries/ 3428

2 blood vessel injury.mp. 192

3 "arter* injur*".mp. [mp=title, abstract, original title, name of substance word, subject heading word, floating sub-heading word, keyword heading word, organism supplementary concept word, protocol supplementary concept word, rare disease supplementary concept word, unique identifier, synonyms] 10238

4 1 or 2 or 3 13190

5 exp Lower Extremity/ 176932

6 exp Iliac Artery/ 14443

7 exp Popliteal Artery/ 9981

8 exp Femoral Artery/ 29857

9 exp Tibial Arteries/ 1736

10 (Iliac or iliofemeral or infrainguinal or femoral or popliteal or infrageniculate or tibial or peroneal or tibioperoneal or "lower extremit*").mp. [mp=title, abstract, original title, name of substance word, subject heading word, floating sub-heading word, keyword heading word, organism supplementary concept word, protocol supplementary concept word, rare disease supplementary concept word, unique identifier, synonyms] 352328

11 5 or 6 or 7 or 8 or 9 or 10 475581

12 4 and 11 2595

13 exp Endovascular Procedures/ 135635

14 exp Stents/ 84580

15 endovascular surgery.mp. 1216

16 (hybrid or endovascular or stent*).mp. [mp=title, abstract, original title, name of substance word, subject heading word, floating sub-heading word, keyword heading word, organism supplementary concept word, protocol supplementary concept word, rare disease supplementary concept word, unique identifier, synonyms] 362275

17 13 or 14 or 15 or 16 437119

18 exp Vascular Surgical Procedures/ 276182

19 femorofemoral bypass.mp. 302

20 artery bypass.mp. 70575

21 femoropopliteal bypass.mp. 758

22 bypass surgery.mp. 23804

23 vein bypass.mp. 2090

24 vascular surgery.mp. 13863

25 19 or 20 or 21 or 22 or 23 or 24 96310

26 (open or bypass* or "open surger*").mp. [mp=title, abstract, original title, name of substance word, subject heading word, floating sub-heading word, keyword heading word, organism supplementary concept word, protocol supplementary concept word, rare disease supplementary concept word, unique identifier, synonyms] 749211

27 18 or 25 or 26 947043

28 17 and 27 169050

29 12 and 28 **551**

**embase <1974 to 2023 january 13>**

1 exp blood vessel injury/ 50368

2 "arter* injur*".mp. 18967

3 1 or 2 53689

4 exp lower limb/ 430200

5 exp iliac artery/ 19090

6 exp popliteal artery/ 8835

7 exp femoral artery/ 36484

8 exp tibial artery/ 3448

9 (Iliac or iliofemeral or infrainguinal or femoral or popliteal or infrageniculate or tibial or peroneal or tibioperoneal or "lower extremit*").mp. [mp=title, abstract, heading word, drug trade name, original title, device manufacturer, drug manufacturer, device trade name, keyword heading word, floating subheading word, candidate term word] 421774

10 4 or 5 or 6 or 7 or 8 or 9 738071

11 3 and 10 7212

12 exp endovascular surgery/ 43999

13 exp stent/ 195541

14 (hybrid or endovascular or stent* or revasculariz*).mp. [mp=title, abstract, heading word, drug trade name, original title, device manufacturer, drug manufacturer, device trade name, keyword heading word, floating subheading word, candidate term word] 630256

15 12 or 13 or 14 631180

16 exp femorofemoral bypass/ 949

17 exp artery bypass/ 4771

18 exp femoropopliteal bypass/ 2267

19 exp bypass surgery/ 366247

20 exp vein bypass/ 1995

21 (open or bypass* or "open surger*").mp. [mp=title, abstract, heading word, drug trade name, original title, device manufacturer, drug manufacturer, device trade name, keyword heading word, floating subheading word, candidate term word] 1053302

22 16 or 17 or 18 or 19 or 20 or 21 1254012

23 15 and 22 174698

24 11 and 23 717

25 exp short term survival/ or exp survival/ or exp survival rate/ or exp survival analysis/ or exp long term survival/ or exp graft survival/ or exp overall survival/ 1328850

26 exp leg amputation/ or exp amputation/ or exp above knee amputation/ or exp below knee amputation/ or exp knee amputation/ or exp limb amputation/ or exp foot amputation/ 50716

27 exp mortality/ or exp wound healing/ or exp fasciotomy/ or exp compartment syndrome/ 1391805

28 25 or 26 or 27 2490594

29 24 and 28 **308**

**cochrane central register of controlled trials for randomized controlled trials:**

vessel injury and Lower Extremity **4**

**Supplementary Table 1. Characteristics of included studies.**

| Study, Year | Country | Recruitment Period | Penetrating percent-% (ET/OSR) | Blunt percent-% (ET/OSR) | Fracture or dislocation | Injury scoring mechanism | scoring in ET/OSR | Type of ET | Type of OSR |
| --- | --- | --- | --- | --- | --- | --- | --- | --- | --- |
| Potter, 2021 ^23^ | USA | 2007-2014 | NA | NA | Partial | ISS | 10 (2-38)/ 10 (4-75) | NA | NA |
| Abdou(P)†, 2021 ^25^ | USA | 2002-2016 | 100/100 | NA | partial | ISS | 17 (13- 23)/ 16 (14-19) | NA | NA |
| Abdou(B)‡, 2021 ^25^ | USA | 2002-2016 | NA | 100/100 | partial | ISS | 32 (22-41)/ 26 (20-38) | NA | NA |
| Degmetich, 2020 ^22^ | USA | 2012-2014 | 29.5/54.2 | 59.0/39.9 | NA | NA | NA | stent | suture, incision, bypass, patch |
| Ratnasekera, 2020 ^30^ | USA | 2013-2017 | 100/100 | NA | NA | ISS | 10.5(10-17)/ 17 (9- 24) | NA | NA |
| Maithel, 2020 ^29^ | USA | 2014-2016 | NA | NA | NA | NA | 10/7 | stent, PTA | suture, incision, bypass, patch |
| Butler, 2019 ^27^ | USA | 2007-2014 | 18.9/40.1 | 46/43.9 | partial | ISS | 9 (4–22)/ 13 (9–17) | stent, PTA | suture, incision, bypass, patch |
| Wahab, 2019 ^24^ | Egypt | 2016-2017 | NA | NA | NA | NA | NA | stent | vein bypass |
| Branco, 2017 ^26^ | USA | 2007-2014 | NA | NA | NA | NA | NA | stent, PTA | suture, incision, bypass, patch |
| Branco, 2014 ^13^ | USA | 2002-2010 | NA | NA | NA | NA | NA | stent, PTA | suture, incision, bypass, patch |
| Dua, 2014 ^28^ | USA | 1998-2011 | NA | NA | NA | NA | NA | stent | bypass, ligations, thrombectomy |
| Banion, 2021 ^42^ | USA | 2007-2018 | NA /35 | NA /65 | partial | ISS | NA / 13+-10 | NA | bypass, patch |
| Jiang, 2021 ^8^ | China | 2012-2020 | 21.7/ NA | 78.3/ NA | NA | ISS | 15.8+-6.2/ NA | stent, PTA | NA |
| Hundersmarck, 2021 ^37^ | USA | 2000-2019 | NA | NA /100 | partial | NA | NA | NA | bypass |
| Georgakarakos, 2021 ^36^ | Greece | 2015-2019 | NA | NA /5/17 | partial | NA | NA | NA | ligations, bypass, patch |
| Asensio, 2020 ^32^ | USA | NA | NA /54/76 | NA /22/76 | partial | NA | NA/21.0 + - 7.7 | NA | bypass |
| Rehman, 2020 ^45^ | Pakistan | 2008-2018 | NA/72.5 | NA/27.5 | partial | NA | NA | NA | bypass |
| Sharrock, 2019 ^12^ | UK | 2001-2014 | NA /100 | NA | NA | ISS | NA / 18.19 | NA | bypass, ligations, thrombectomy, patch |
| Prieto, 2019 ^44^ | USA | 2007-2014 | NA | NA | NA | NA | NA | NA | NA |
| Magnotti (P)†, 2020 ^40^ | USA | 1996-2015 | NA /100 | NA | partial | ISS | NA /9 (9-9) | NA | NA |
| Magnotti (B)‡, 2020 ^40^ | USA | 1996-2015 | NA | NA /100 | partial | ISS | NA/9 (9- 17) | NA | NA |
| Mousa, 2018 ^41^ | Egypt | 2008-2015 | NA | NA | NA | NA | N/A | NA | vein bypass |
| Şahin, 2018 ^46^ | Turkey | 2014-2016 | NA | NA | NA | NA | NA | NA | suture, bypass |
| Kufner, 2015 ^39^ | Germany | 2010-2013 | 100/NA | NA | NA | NA | NA | stent | NA |
| Lang, 2015 ^10^ | Austria | 1999-2009 | NA /29/64 | NA /35/64 | partial | ISS | NA | NA | suture, bypass, patch |
| Dua, 2014 ^34^ | USA | 2003-2007 | NA /96 | NA | partial | ISS | NA /18.7 | NA | NA |
| Sciarretta,2014 ^47^ | USA | 2006- 2011 | NA /14/18 | NA /4/18 | partial | ISS | NA / 16.5+-4.4 | NA | suture, bypass, patch |
| Bernhoff, 2013 33 | Sweden | 1987- 2011 | NA | NA | all | NA | NA | NA | NA |
| Dua, 2012 ^35^ | USA | 2006- 2011 | NA | NA | partial | NA | NA | NA | suture, ligations, thrombectomy, bypass, patch |
| Trellopoulos, 2012 ^48^ | Greece | 2003- 2009 | NA | NA | NA | NA | NA | stent, embolization | NA |
| Pourzand, 2010 ^43^ | Iran | 2004-2009 | NA | NA /100 | partial | NA | NA | NA | suture, thrombectomy, bypass |
| Huynh, 2006 ^38^ | USA | 2001-2006 | NA | NA / 88.6 | partial | ISS | NA/17.4+-10.6 | NA | bypass |
| White, 2006 ^49^ | USA | 1997-2003 | NA | NA | NA | NA | NA | stent | NA |
| Aksoy, 2005 ^31^ | Turkey | 2001-2004 | 100/ NA | NA | NA | NA | NA | embolization | NA |
| Data are expressed as mean +- standard deviation or median (range) or mean; NA = not available; † penetrating injury; ‡ blunt injury; * All Patients with knee replacement; ET=endovascular therapy; OSR=open surgical repair; PTA=percutaneous transluminal angioplasty | | | | | | | | | |

**Supplementary Table 2. Assessment for Observational Studies (Robins-I Tool).**

|  | | | | | | | |
| --- | --- | --- | --- | --- | --- | --- | --- |
| **Study** | **Confounding** | **Selection** | **Measurement of interventions** | **Deviations from intended interventions** | **Missing data** | **Measure-ment of data** | **Selection of the reported result** |
| **Potter 2021** | M | M | L | M | L | M | L |
| **Abdou 2021** | M | M | M | M | M | M | L |
| **Ratnasekera 2020** | M | M | M | M | M | M | L |
| **Maithel 2020** | M | M | L | M | S | M | S |
| **Degmet-ich 2020** | M | M | M | M | S | M | L |
| **Butler 2019** | M | M | M | M | L | M | L |
| **Wahab 2019** | S | S | M | S | M | M | M |
| **Branco 2017** | M | M | M | M | M | M | M |
| **Dua 2015** | S | S | M | S | M | M | L |
| **Branco 2014** | M | M | M | M | L | M | M |

M= moderate; L=low; S= Serious

**Supplementary Table 3. Major components of the 18-item tool developed by Carmen Moga and colleagues for assessing case series.**

| **Major components** | **Judgment** |
| --- | --- |
| 1. Is the hypothesis/aim/objective of the study clearly stated? | 1. Yes, Unclear, No |
| 2. Are the characteristics of the participants included in the study described? | 2. Yes, Partially reported, No |
| 3. Were the cases collected in more than one center? | 3. Yes, Unclear, No |
| 4. Are the eligibility criteria (ie, inclusion and exclusion criteria) for entry into the study clearly stated? | 4. Yes, Partially reported, No |
| 5. Were participants recruited consecutively? | 5. Yes, Unclear, No |
| 6. Did participants enter the study at a similar point in the disease? | 6. Yes, Unclear, No |
| 7. Was the intervention of interest clearly described? | 7. Yes, Partially reported, No |
| 8. Were additional interventions (cointerventions) reported in the study? | 8. Yes, Unclear, No |
| 9. Are the outcome measures established a priori? | 9. Yes, Partially reported, No |
| 10. Were the relevant outcomes measured with appropriate objective or subjective methods? | 10. Yes, Unclear, No |
| 11. Were the relevant outcomes measured before and after the intervention? | 11. Yes, Unclear, No |
| 12. Were the statistical tests used to assess the relevant outcomes appropriate? | 12. Yes, Unclear, No |
| 13. Was the length of follow-up reported? | 13. Yes, Unclear, No |
| 14. Was the loss to follow-up reported? | 14. Yes, Unclear, No |
| 15. Does the study provide estimates of the random variability in the data analysis of relevant outcomes? | 15. Yes, Unclear or partially reported, No |
| 16. Are the adverse events related with the intervention reported? | 16. Yes, Partially reported, No |
| 17. Are the conclusions of the study supported by results? | 17. Yes, Partially reported, No |
| 18. Are both competing interests and sources of support for the study reported? | 18. Yes, Partially reported, No |

**Supplemental Table 3.** Results of quality assessment of case series by 18-item tool

| Author | Year | 1 | 2 | 3 | 4 | 5 | 6 | 7 | 8 | 9 | 10 | 11 | 12 | 13 | 14 | 15 | 16 | 17 | 18 |
| --- | --- | --- | --- | --- | --- | --- | --- | --- | --- | --- | --- | --- | --- | --- | --- | --- | --- | --- | --- |
| Banion | 2021 | Y | Y | Y | Y | Y | N | Y | Y | Y | Y | Y | Y | Y | P | Y | Y | Y | Y |
| Jiang | 2021 | Y | Y | N | Y | Y | Y | Y | Y | Y | Y | Y | Y | Y | Y | Y | Y | Y | Y |
| Georgakarak-os | 2021 | Y | Y | N | N | Y | Y | Y | Y | Y | Y | Y | Y | Y | Y | N | Y | Y | Y |
| Hundersmar-ck | 2021 | Y | Y | Y | Y | Y | Y | Y | Y | N | Y | Y | Y | Y | Y | N | Y | Y | Y |
| Asensio | 2020 | Y | P | N | Y | N | N | Y | Y | Y | Y | Y | Y | Y | N | N | Y | Y | Y |
| Rehman | 2020 | Y | Y | N | Y | Y | Y | Y | Y | Y | Y | Y | Y | P | N | N | Y | Y | Y |
| Sharrock | 2019 | Y | Y | Y | Y | Y | Y | Y | Y | Y | Y | Y | Y | Y | Y | N | Y | Y | Y |
| Magnotti | 2020 | Y | Y | N | Y | U | Y | Y | Y | Y | Y | Y | Y | Y | Y | Y | Y | Y | Y |
| Prieto | 2019 | Y | Y | Y | U | U | Y | Y | Y | Y | Y | Y | Y | N | U | Y | Y | Y | Y |
| Mousa | 2018 | Y | Y | Y | U | Y | Y | Y | U | Y | Y | N | P | N | U | U | Y | Y | Y |
| Şahin | 2018 | Y | Y | N | U | U | U | Y | N | Y | U | N | Y | N | N | N | Y | Y | Y |
| Kufner | 2015 | Y | Y | N | Y | Y | Y | Y | Y | Y | Y | Y | Y | Y | N | U | Y | Y | Y |
| Lang | 2015 | Y | Y | N | U | Y | Y | Y | Y | Y | Y | U | Y | U | U | N | Y | Y | Y |
| Sciarretta | 2014 | Y | Y | N | Y | Y | Y | Y | U | Y | Y | Y | Y | N | N | U | Y | Y | Y |
| Dua | 2014 | Y | Y | Y | Y | U | Y | N | N | Y | Y | N | Y | U | U | N | Y | Y | Y |
| Bernhoff | 2013 | Y | Y | Y | N | U | U | Y | U | Y | Y | Y | Y | Y | Y | N | Y | Y | Y |
| Dua | 2012 | Y | Y | Y | N | U | U | Y | U | Y | Y | Y | Y | Y | Y | U | Y | Y | Y |
| Trellopoulos | 2012 | Y | Y | N | Y | Y | Y | Y | N | Y | Y | Y | Y | Y | Y | U | N | Y | Y |
| Pourzand | 2010 | Y | Y | N | U | U | U | Y | Y | Y | Y | N | Y | Y | Y | N | Y | Y | Y |
| Huynh | 2006 | Y | Y | N | Y | U | N | Y | N | Y | Y | Y | Y | Y | Y | N | Y | Y | Y |
| White | 2006 | Y | Y | N | U | U | Y | Y | U | Y | Y | Y | Y | N | N | U | Y | Y | Y |
| Aksoy | 2005 | Y | Y | N | N | N | U | Y | U | Y | Y | N | U | N | U | U | P | Y | Y |

N, NO, not reported; P, Partially reported; U, Unclear; Y, Yes, fully reported. Items 1 to 18 indicate 18 components of quality assessment for case series, shown in Supplemental Table 3.


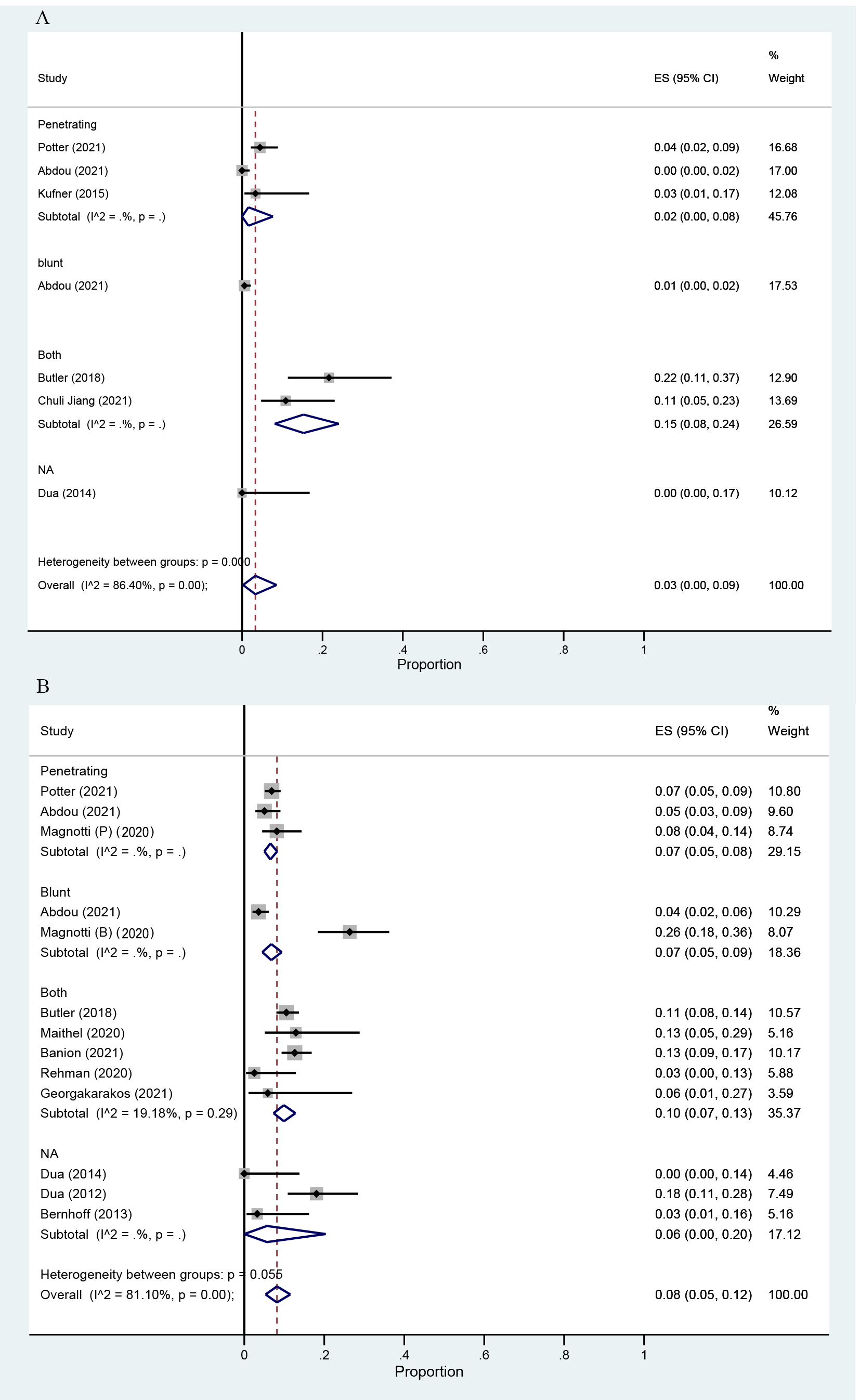


Supplemental Figure 1. The pooled estimate for amputation in ET (A) and OSR (B) in adults with traumatic lower extremity arterial injury. ES=estimate proportions; CI=confidence interval; ET=endovascular therapy; OSR=open surgical repair.


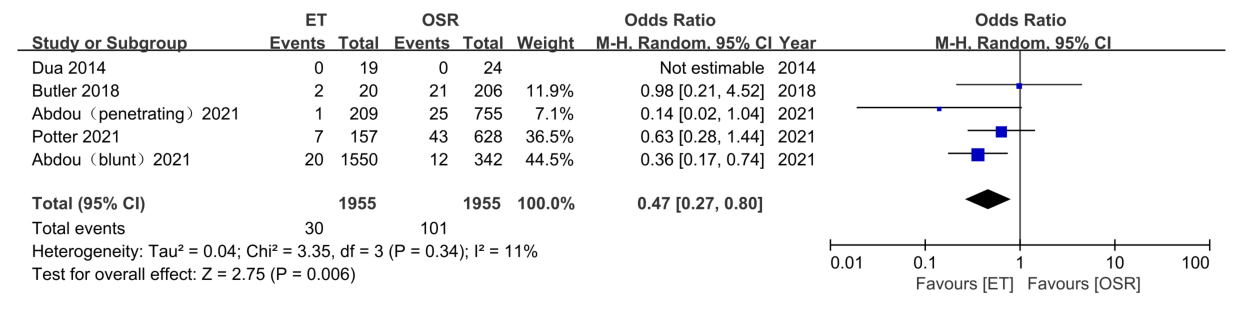


Supplementary Figure 1C. ET reduce the incidence of amputation in adults using all available data. M-H = Mantele-Haenszel; CI = confidence interval; ET=endovascular therapy; OSR=open surgical repair.


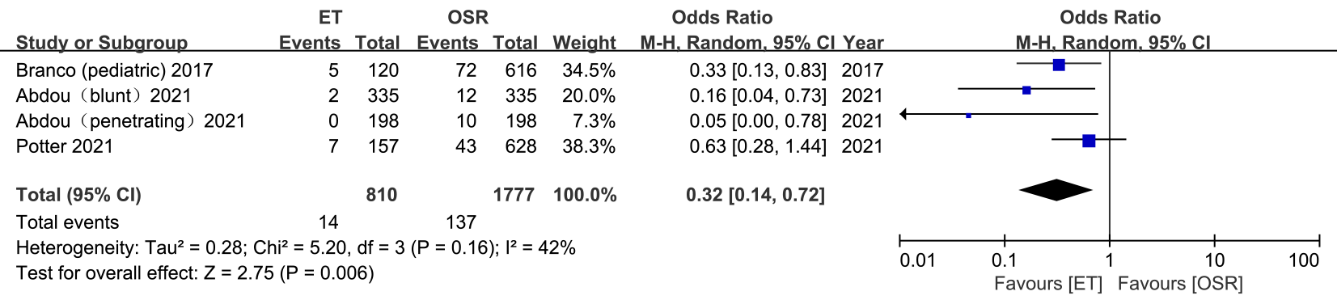


Supplementary Figure 1D. ET reduce the incidence of amputation using propensity score-matched data. M-H = Mantele-Haenszel; CI = confidence interval; ET=endovascular therapy; OSR=open surgical repair.


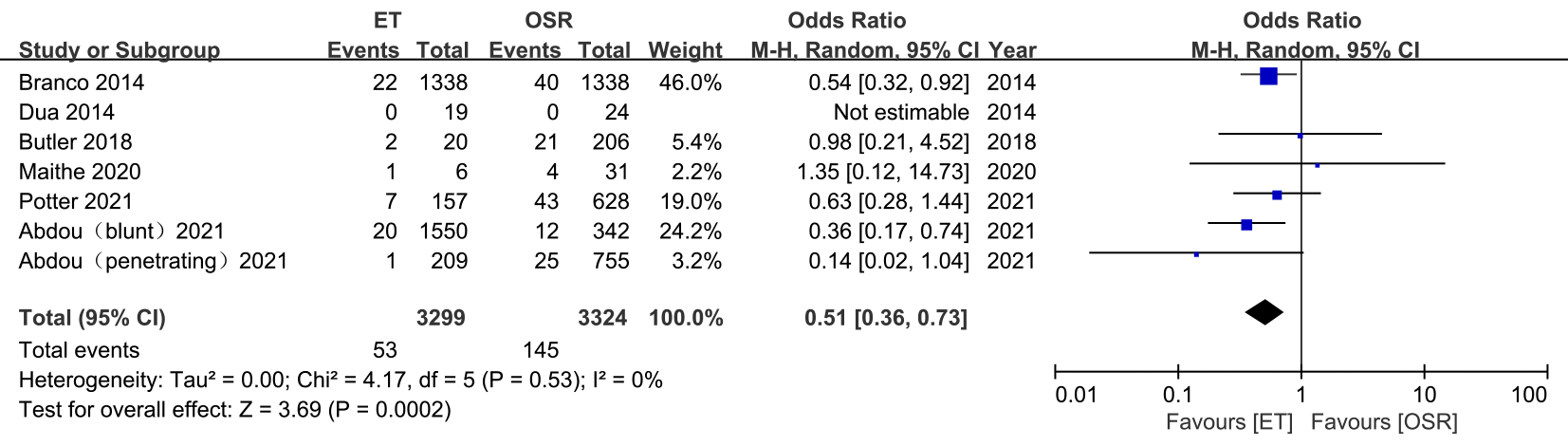


Supplementary Figure 1E. ET reduce the incidence of amputation using all available data. M-H = Mantele-Haenszel; CI = confidence interval; ET=endovascular therapy; OSR=open surgical repair.


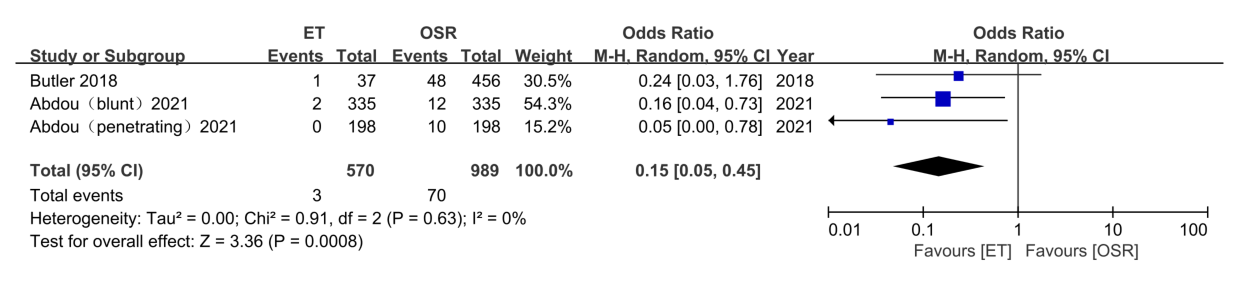


Supplementary Figure 1F. ET reduce the incidence of amputation in patients with Iliac or femoral arterial injury. M-H = Mantele-Haenszel; CI = confidence interval; ET=endovascular therapy; OSR=open surgical repair.


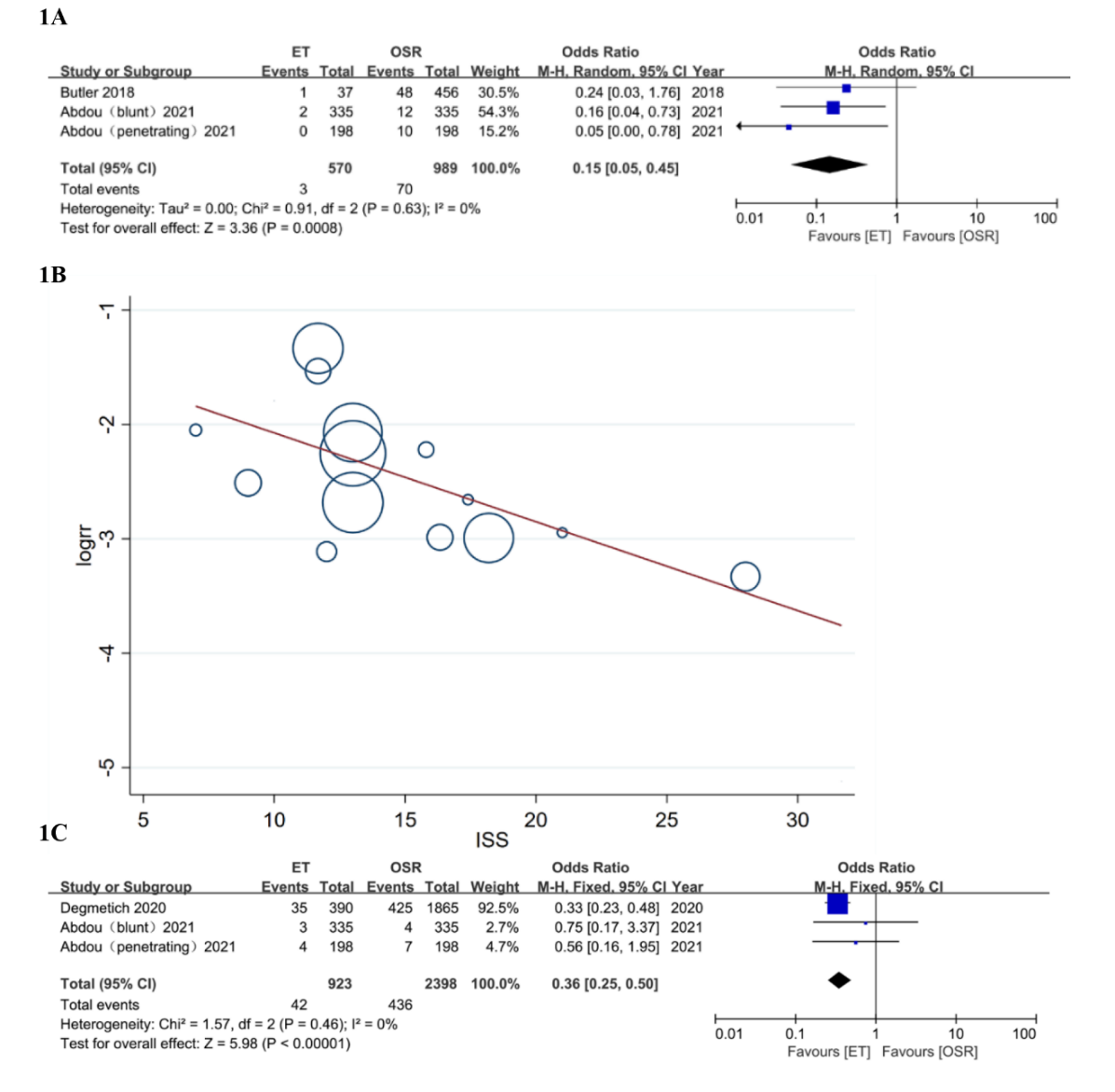


Supplementary Figure 1G. Bubble plots indicating meta-regression of ISS with amputation. ES: estimate proportions.


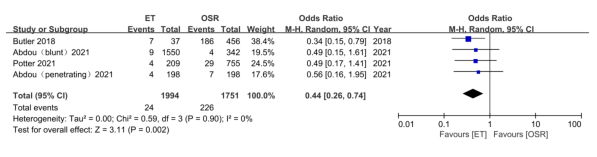


Supplementary Figure 2A. ET reduce the incidence of fasciotomy or compartment syndrome using all available data. M-H = Mantele-Haenszel; CI = confidence interval; ET=endovascular therapy; OSR=open surgical repair


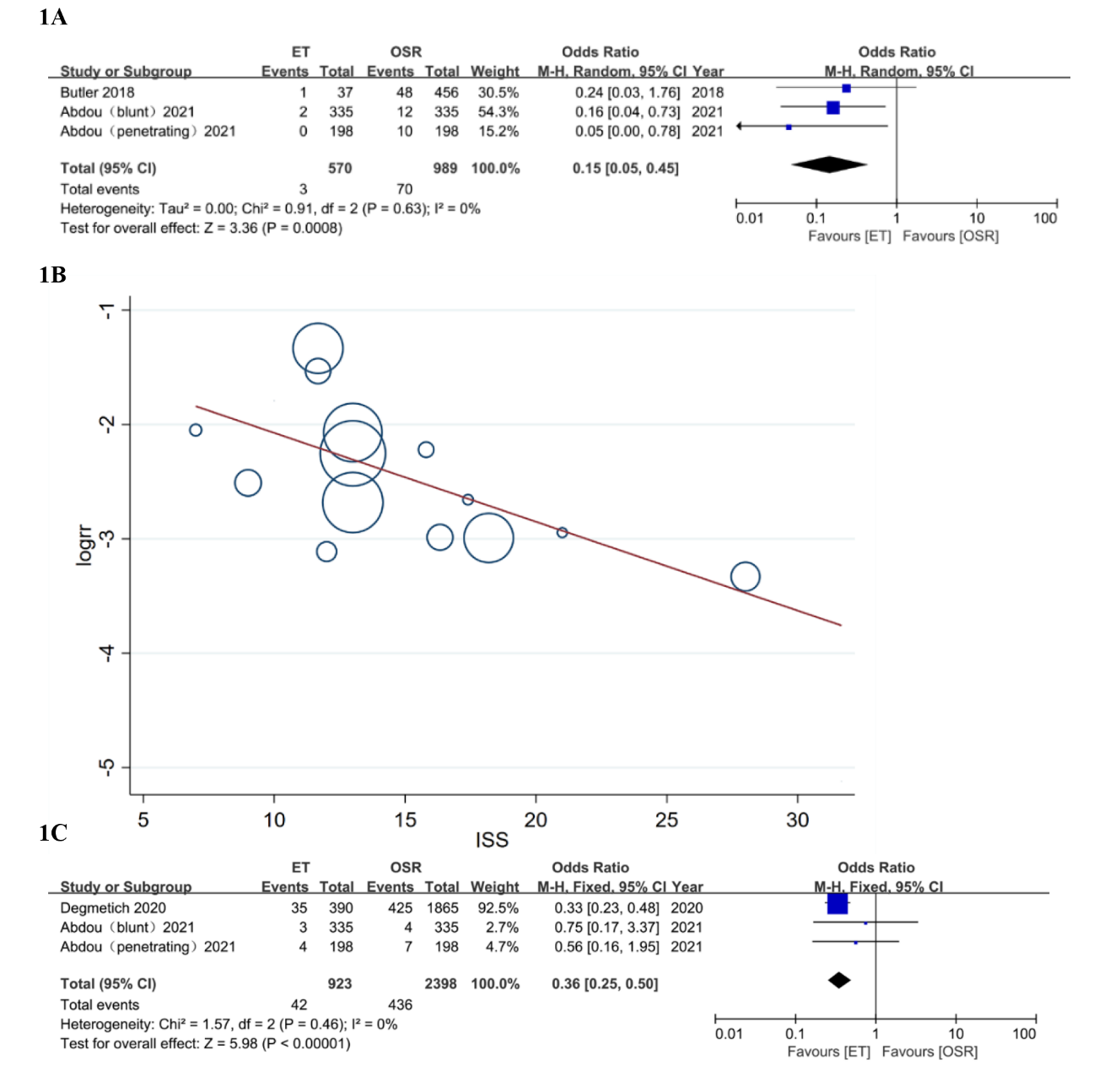


Supplementary Figure 2B. ET reduce the compartment syndrome than OSR. M-H = Mantele-Haenszel; CI = confidence interval; ET=endovascular therapy; OSR=open surgical repair.


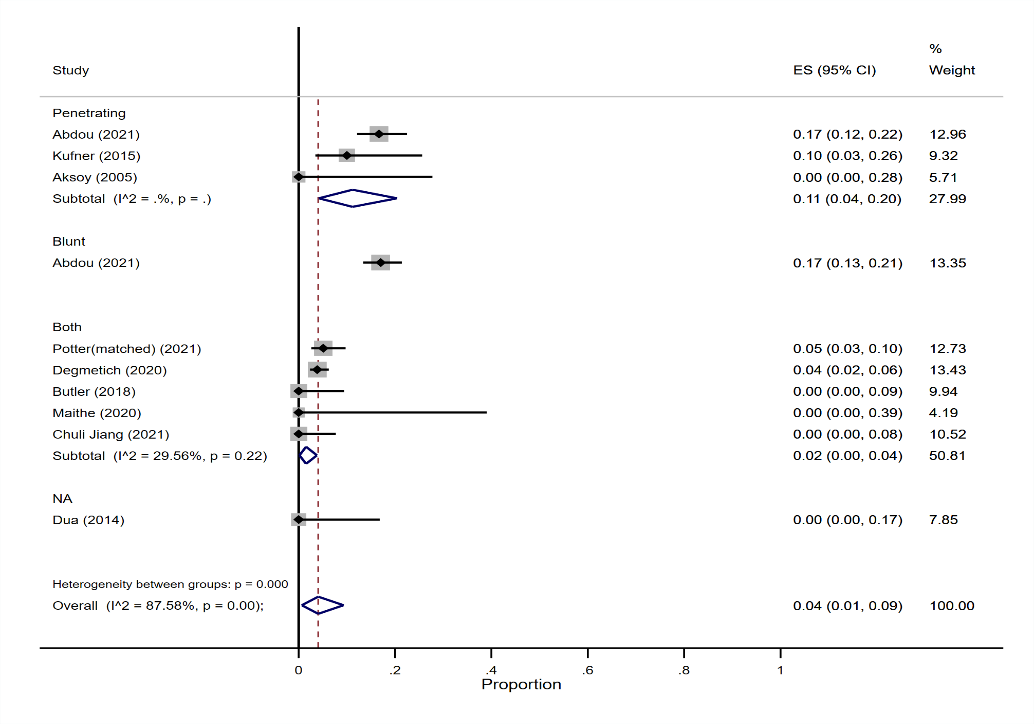


Supplementary Figure 3A. The pooled estimate for mortality in ET. ES = estimate proportions; CI = confidence interval; ET=endovascular therapy.


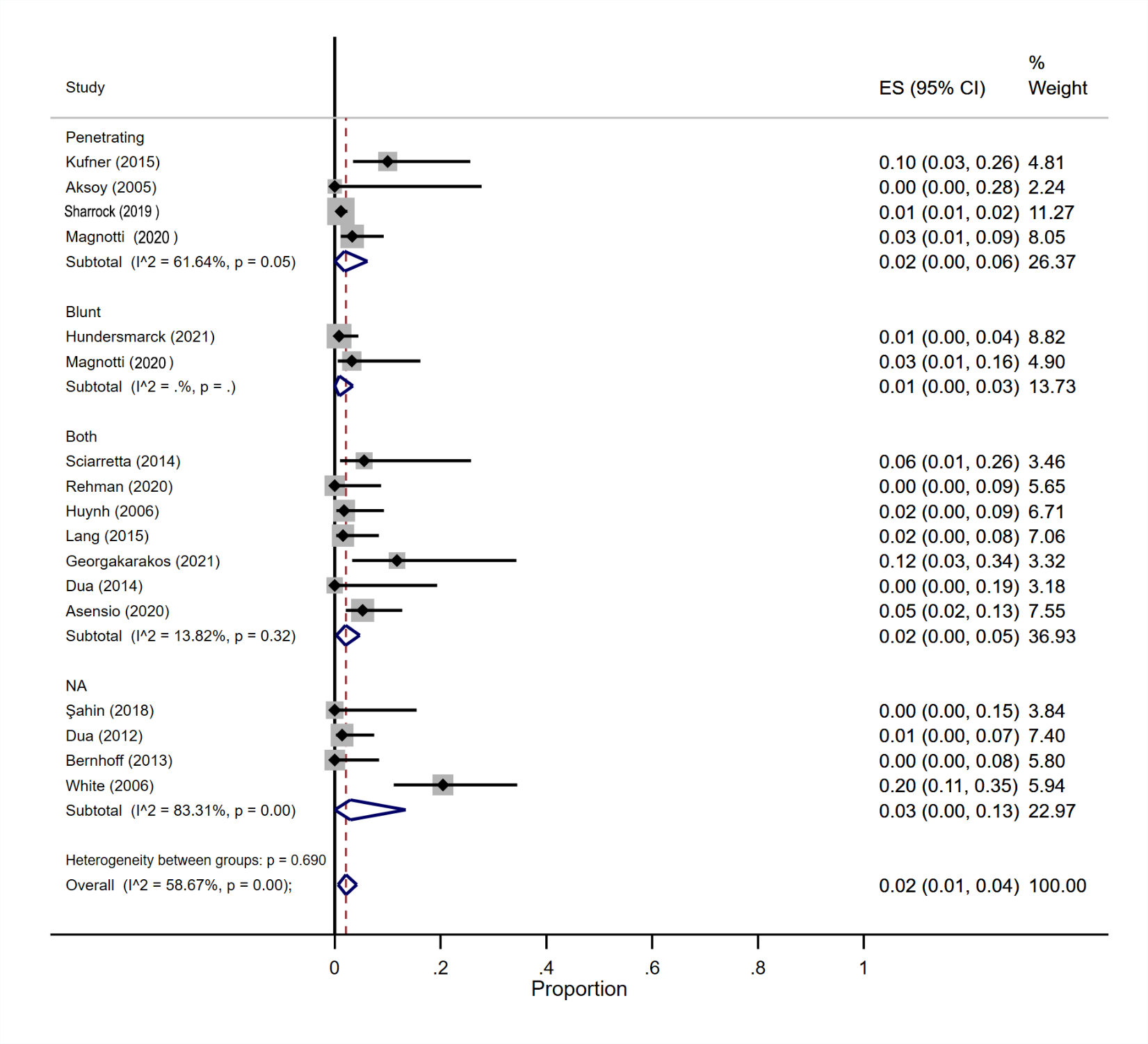


Supplementary Figure 3B. The pooled estimate for mortality in OSR. ES = estimate proportions; CI = confidence interval; OSR=open surgical repair.


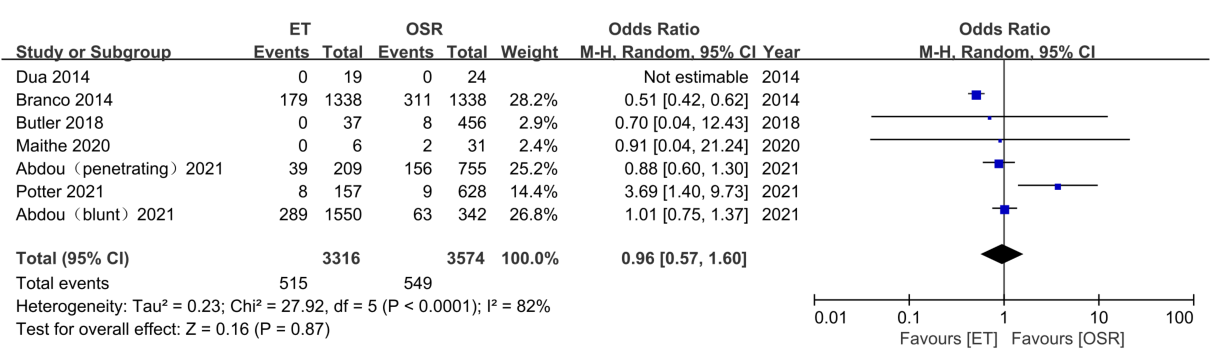


Supplementary Figure 3C. ET can not reduce the incidence of all-cause mortality using all available data. M-H = Mantele-Haenszel; CI = confidence interval; ET=endovascular therapy; OSR=open surgical repair.


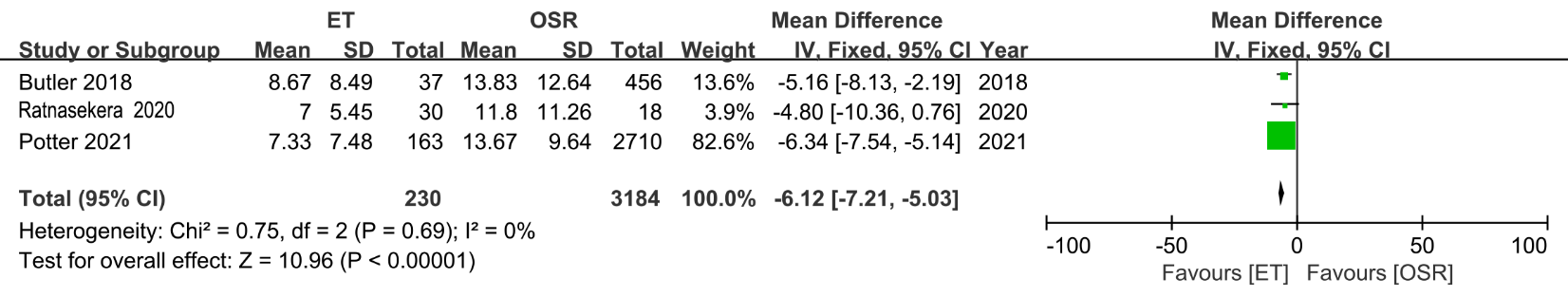


Supplementary Figure 3D. ET decrease the length of stay in patients with penetrating arterial injury. IV=inverse variance; CI = confidence interval; ET=endovascular therapy; OSR=open surgical repair.
